# Quantifying States and Transitions of Emerging Postural Control for Children Not Yet Able to Sit Independently

**DOI:** 10.3390/s23063309

**Published:** 2023-03-21

**Authors:** Patricia Mellodge, Sandra Saavedra, Linda Tran Poit, Kristamarie A. Pratt, Adam D. Goodworth

**Affiliations:** 1 Department of Electrical and Computer Engineering, College of Engineering, Technology, and Architecture, University of Hartford, West Hartford, CT 06117, USA; 2 Physical Therapy Program, College of Health Sciences, Western University of Health Sciences-Oregon, Lebanon, OR 97355, USA; ssaavedra@westernu.edu; 3 Hartford Hospital, Hartford, CT 06106, USA; tran.linda10@gmail.com; 4 Department of Rehabilitation Sciences, College of Education, Nursing and Health Professions, University of Hartford, West Hartford, CT 06117, USA; kpratt@hartford.edu; 5 Department of Kinesiology, Westmont College, Santa Barbara, CA 93108, USA; agoodworth@westmont.edu

**Keywords:** motor control, accelerometer, cerebral palsy, assessment, trunk, biomechanical algorithm, postural development model

## Abstract

Objective, quantitative postural data is limited for individuals who are non-ambulatory, especially for those who have not yet developed trunk control for sitting. There are no gold standard measurements to monitor the emergence of upright trunk control. Quantification of intermediate levels of postural control is critically needed to improve research and intervention for these individuals. Accelerometers and video were used to record postural alignment and stability for eight children with severe cerebral palsy aged 2 to 13 years, under two conditions, seated on a bench with only pelvic support and with additional thoracic support. This study developed an algorithm to classify vertical alignment and states of upright control; Stable, Wobble, Collapse, Rise and Fall from accelerometer data. Next, a Markov chain model was created to calculate a normative score for postural state and transition for each participant with each level of support. This tool allowed quantification of behaviors previously not captured in adult-based postural sway measures. Histogram and video recordings were used to confirm the output of the algorithm. Together, this tool revealed that providing external support allowed all participants: (1) to increase their time spent in the Stable state, and (2) to reduce the frequency of transitions between states. Furthermore, all participants except one showed improved state and transition scores when given external support.

## 1. Introduction

Postural control creates the foundation for upright activity such as sitting and mobility, thus it is not surprising that prognosis for motor skills is limited for children with deficits in trunk control [1,2,3]. Failure to develop trunk control cascades into a plethora of secondary complications and limitations, including increased risk for musculoskeletal deformities such as scoliosis [4], hip dysplasia [5], osteoporosis [6], fractures [7], and diminished growth [8]. These complications place them at increased health risks for respiratory illness [9,10,11], pressure wounds [12,13], joint contractures [14], and complex surgical interventions for scoliosis [4] or hip dysplasia [15,16]. Lack of trunk control impairs the child’s ability to play, interact with the environment, and freely use their hands, which leads to cognitive challenges due to missed learning opportunities [17].

Most theoretical models of postural development consider the trunk as if it were a single segment [18,19,20,21,22], and thus lead to developmental assessments that examine posture control in an all or none fashion. There are limited outcome measures that are specifically designed for those who have not achieved independent sitting or mobility. Standardized assessments such as the Gross Motor Function Measure (GMFM) [23], or Peabody Developmental Motor Scales (PDMS) [24] often have floor effects for non-ambulatory children and lack the granularity necessary to document small improvements in posture control. These assessments document the child’s ability to support the entire trunk upright either by propping on arms or hands free or evaluate the child’s ability to lift their head in supine or prone positions. In short, these assessments of postural progression lack the granularity and precision to document the process by which children first learn to attain and maintain upright alignment. This is an important socioeconomic problem as populations with deficits in trunk control have the greatest burden of care and need for improved services [25].

One example of an assessment with more granularity and precision is the Segmental Assessment of Trunk Control (SATCo) [26], which is based on a multi-segment model of the trunk and the historical evidence of a cephalocaudal progression of motor control [27,28]. The SATCo allows documentation of the child’s level of static, active, and reactive control across seven regions of the trunk while positioned vertically [26]. The SATCo not only offers a precise assessment of partial trunk control, but it also challenges clinicians and researchers to reconsider the single-segment model and include the impact of specific segmental levels of control on function. The SATCo offers a more precise evaluation of body structure and function that provides information about the child’s current level of upright control and has been shown to be related to functional status. One disadvantage of the SATCo is that training is required, and it takes at least two trained testers to administer the assessment. While SATCo provides more specific information about the current level of control, large gaps remain with respect to understanding mechanisms used during the development of upright control, and, thus, little is known about how to design effective interventions that will help a child change their current level of trunk control [29,30]. Quantitative measures of postural behavior using varying levels of trunk support might provide increased granularity to examine and understand underlying sensory motor mechanisms.

Postural sway is an example of a postural behavior that is quantified in adults and children who have trunk control. Postural sway is a measure of the unconscious, small movements that happen around the body’s center of gravity. Changes in amplitude, velocity, or frequency content of postural sway are amongst the many experimental metrics that are used to quantify postural stability or control [31]. Postural sway is traditionally collected with force plates (e.g., center of pressure [31]) and/or with body worn kinematic markers or sensors that provide a direct and detailed measure of body segment movements [32,33,34].

Child development researchers investigate postural sway similar to studies in adults by examining amplitude and velocity of postural sway for brief seconds before infants collapse or fall [19,20,35]. Similar measures of postural sway are used to evaluate infants who are able to remain upright [22,36] or positioned in a reclined seat and given visual or motor perturbations [37,38]. However, the parameters used for these studies are based on mature postural sway behaviors and not translatable in children with deficits in trunk control.

In 2012 Saavedra and colleagues [39] completed a longitudinal study in typical infants that characterized four stages of upright control in infants based on patterns of postural behavior that occur prior to development of adult-like postural sway. The stage descriptions were based on video review of postural behavior when external pelvic support was provided to typical infants. At the youngest ages (2–3 months) when infants are held vertically, the unsupported portion of their trunk collapses forward, sideways, or backwards until it reaches a supporting surface or end of available range (named the Collapse stage). The next stage (Rise and Fall stage) is seen in infants at 3–4 months of age. During this period infants exhibit ballistic muscle responses, primarily of trunk extensors, in an attempt to overcome gravity. However, the infants do not yet have the control necessary to refine the muscle activation, and they either overshot vertical and fell backwards or came partially upright and fell forwards. The Rise and Fall stage is followed at 4–6 months of age by a stage of continuous muscle activation and modulation resulting in a teeter-totter kind of wobbling pattern (named the Wobble stage). This pattern can be observed with an infant leaning forward using primarily the trunk extensors or full range with the infant alternating activation between trunk flexors and extensors as they wobble forwards and backwards from midline. The final stage (Stable stage) becomes apparent at 7–8 months of age and is similar to adult postural sway. This more stable postural sway pattern is evident by smaller amplitude and velocity of sway around vertical alignment. Using histograms of anterior-posterior position in space across the full 3-min trial provides a method for generalized classification of each infant’s stage of control (e.g., skewed away from midline for Collapse; bimodal distribution for Rise and Fall behavior; flatter, more uniform bell-shaped distribution for Wobble; and a narrow bell-shaped distribution for Stable) [39]. These same four stages (Collapse, Rise and Fall, Wobble, and Stable) were subsequently demonstrated in children with moderate to severe cerebral palsy [40]. Based on the Saavedra studies [39,40], primary stages can be distinguished for most infants and children using histograms. However, it is not possible with histograms to distinguish between multiple behaviors within a 3-min interval or to quantify amount of time spent in each behavior. Saavedra and colleagues noted variability in postural behaviors of infants within the 3-min trial [39]. For example, histograms might indicate that an infant is classified as Collapse, but video reveals attempt to Rise one or two times, or Rise and Wobble for a few seconds before Falling. Increased granularity beyond what is available in histograms is needed to examine how postural stages change from moment to moment or at hourly, daily, or monthly time scales and which patterns of change lead to achieving upright control.

Goodworth and Saavedra demonstrate that external support can be used to study postural responses to perturbation prior to the emergence of upright control for sitting [41,42]. These studies evaluate postural behaviors based on sensorimotor feedback modeling and do not include examination of postural stages. There currently are no outcome measures with the granularity to quantify dynamic fluctuations in postural behavior in a continuous manner across different time scales (from seconds to months) for infants or children who lack postural control and independent sitting ability. Such a method is critically needed to examine the effect of interventions, positioning equipment, and daily activity on the emergence of upright control. Furthermore, to understand the process behind the development of vertical posture, the assessments performed need to take these posture behavioral stages into account. No previous studies have examined posture development in this manner. This study proposes a method to quantify these postural behaviors.

The purpose of this study is threefold. First, to determine if wearable sensors are a viable means to collect kinematic data showing orientation and stability of the head and trunk. Second, to create an algorithm that uses kinematic data to differentiate and quantify postural stages moment to moment. Third, to examine transitions in postural stages across time and with different levels of support. For this purpose, a cross sectional design with children who have cerebral palsy using supported and unsupported postural conditions was implemented. Previous research has shown that external support can be used to study emergence of upright control in populations with underdeveloped or delayed sitting ability [41,43]. Use of two support levels provided an opportunity to explore change in postural behavior and assess the ability of the algorithm to differentiate between postural conditions.

## 2. Materials and Methods

### 2.1. Participants

A repeated measures study design was used to evaluate the efficacy of using wearable sensors to determine stages of postural control in eight children, between the ages of 23 months and 13 years old (with moderate to severe neuromotor dysfunction and deficits in sitting postural control). Children were recruited from the database for the Pediatric Balance Lab at the University of Hartford. The participant group was chosen to be representative of the wide range of motor capacities seen in children with cerebral palsy. Children from 2–14 years of age were accepted because trunk control is related to severity of CP, and not related to age [44]. All children were classified as Gross Motor Function Classification Scale (GMFCS) [45] level IV or V. Of the eight participants, one child presented with ataxia, two children had dyskinesia, and five had spastic cerebral palsy [46,47]. Prior to working with the child, a signed written informed consent was obtained from the child’s parent. This study was approved by the University of Hartford Institutional Review Board (IRB). Table 1 provides demographics regarding the participants in this study.

### 2.2. Experimental Methods

**Figure 1:**
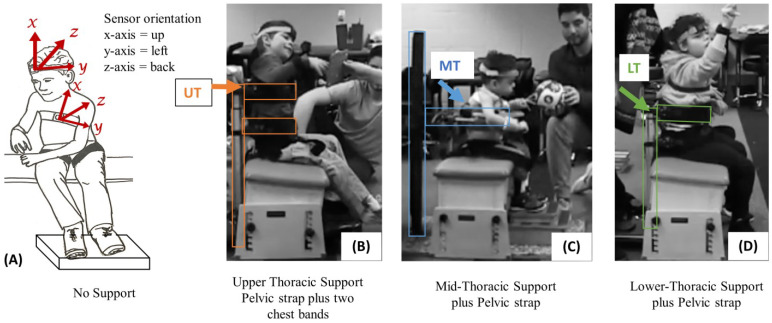
Sensor placement, orientation, and support conditions. Sensors were securely strapped to the children’s heads and chests. Pelvic support stability strapping was included for all levels of support (**A**), and external support was provided at upper (UT), mid (MT), or lower thoracic (LT) regions based on each child’s segmental level of trunk control. The black torso bands are connected to a vertical bar behind the child to provide support at the appropriate level. Trunk supports shown are (**B**) Meerkat with 2 torso bands at UT and LT, (**C**) Custom trunk support device with one band at MT, and (**D**) Meerkat with 1 band at LT.

Video and accelerometry data were collected simultaneously while children were seated on a bench with either (1) pelvic support [26] or (2) with pelvic and trunk support at the child’s level of control as determined by the SATCo. Video recordings were included to disambiguate and verify that the algorithm was adequately quantifying postural behaviors. Two Canon FS400 camcorders (Canon Inc., Tokyo, Japan) on tripods were placed directly anterior and lateral to the child. The cameras recorded at a rate of 30 frames per second. APDM Opal sensors (APDM Wearable Technologies Inc., Portland, OR, USA) were placed on the anterior head and trunk using soft Velcro bands (Figure 1, left image). These sensors have triaxial accelerometers, gyroscopes, and magnetometers, which can, respectively, quantify acceleration, angular velocity, and orientation in space. The sampling rate of the accelerometers was 128 Hz. Simultaneous kinematic data synchronously streamed from the Opal sensors strapped to the child’s head and torso were time matched to video data collected from the two cameras.

During both trials, the participants were seated on an adjustable bench with pelvic strapping for stability [26]. During the first trial children were provided additional external trunk support at the lowest level of the trunk where control was demonstrated during SATCo (e.g., upper thoracic (UT), mid thoracic (MT), or lower thoracic (LT)). During the second trial all children had only pelvic support (No Support condition). See Figure 1. Pelvic strapping was used as described in the Butler manuscript (SATCo reliability) for all participants. In addition, circumferential torso support was provided from behind the child. For the first 2 children a custom support device was used. For the last 6 children a commercially available stander (R82 Meerkat, Etac AB, Kista, Sweden) was used off-label to provide solid vertical trunk support in a seated position. The stander was locked in place behind the child. This provided solid circumferential support. The torso straps on the Meerkat were easier to apply and adjust and more comfortable for children than the custom support. The Meerkat stander was not designed for this purpose; however, it worked well under close supervision for the purposes of this study. Depending on the height of the child, one or two adjustable bands were placed around the child’s trunk to support and stabilize the spine below the level of support.

**Figure 2:**
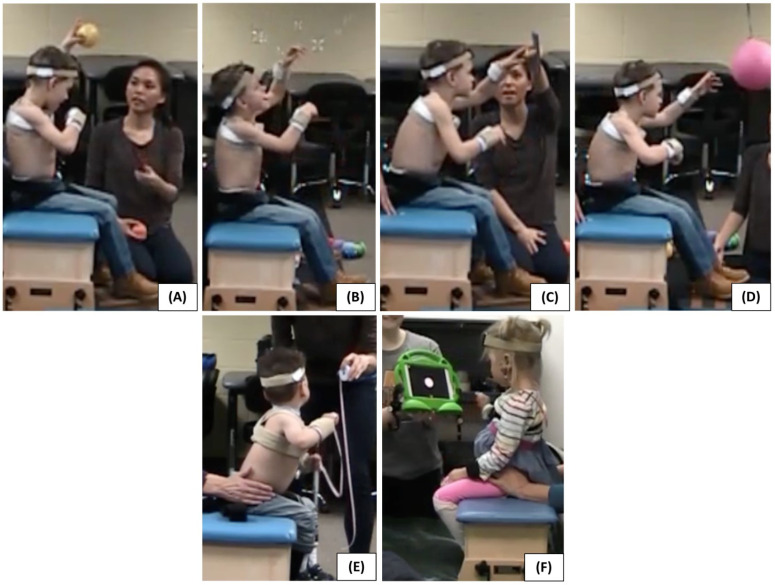
Activities used to facilitate reaching and upright posture. (**A**) Hitting a suspended ball, (**B**) reaching for and popping bubbles, (**C**) placing and removing pegs from a pegboard, and (**D**) reaching for and throwing small balls. Alternative activities were used for children who did not find these four activities motivating, e.g., (**E**) reaching for audiovisual toys and (**F**) reaching for and pulling on a retractable tape measure.

Different activities were used to encourage dynamic balance with unilateral and bilateral arm movements for a total of 12 min. The primary activities included playing with a suspended rubber ball, placing and removing pegs from a peg board, reaching for and throwing plastic balls, or reaching for bubbles (Figure 2). After the first trial, a 5–10-min rest break was given to the child. For the second trial, the child was asked to perform the same tasks with only the pelvic strapping support (without the segmentally adaptive trunk support). The second trial for each child consisted of the same activities, in the same order, and for the same amount of time as their first trial. For safety, a therapist sat behind the participant and guarded the child from injury in the event of rapid movements. When needed, brief support was given to the upper trunk to help the child return to an upright position.

### 2.3. Algorithm Development

**Figure 3:**
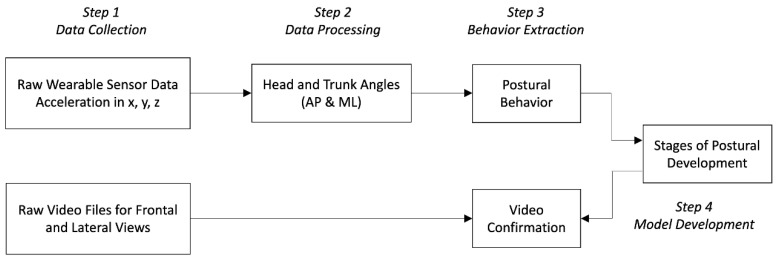
Steps used to process accelerometry data into postural development stage.

The data processing steps are shown in Figure 3. In this study, the process started by using two forms of sensing technology, accelerometers and video, and the final output of the process described the child’s stage of postural development. The algorithm for determining postural behavior (step 3) used a time series of head and trunk angles as input, so it is independent of how the head and trunk angles were quantified. Similarly, the algorithm for determining the stage of postural development (step 4) used a time series of postural behaviors as input, and so is independent of how the postural behavior was generated.

### 2.4. Data Processing

The raw data from the wearable sensors were obtained from the *x*, *y*, and *z* axes of the accelerometer and processed using custom MATLAB scripts. The sampling rate of the sensors was 128 Hz. The data were filtered by means of a 12th order type 2 low pass Chebyshev filter with a 2 Hz bandwidth and 40 dB of stopband attenuation. This filtering step effectively eliminated linear acceleration and noise, leaving only the gravitation component of the acceleration along each axis. This filter type and its parameters were found to give the best performance in terms of removing jerky movements and noise from the signal while maintaining the slower movement patterns of interest.

From the filtered *x*, *y*, and *z* accelerations, the orientation angles of the head and the trunk in the anterior-posterior (AP) and mediolateral (ML) planes were calculated using these formulas:

$$\theta_{ML}=-\mathrm{atan}2\left(y,-x\right)$$

$$\theta_{AP}=-\mathrm{atan}2\left(z,-x\right)$$

These formulas applied if the sensors were upright and the angles were not close to ±90°. However, to account for inverted positions (e.g., positive values for the *x* axis acceleration) and singularities in the atan2 function at ±90°, the following modification was used:

$$\theta_{ML}=\{\begin{array}{ll} \mathrm{atan}2\left(y,x\right), & \mathrm{if}\;\left(x>0\right)\wedge \left(\left|z\right|>\left|y\right|+{\mathrm{acc}}_{thresh}\right)\\ 0, & \mathrm{if}\;\left(\left|x\right|<{\mathrm{acc}}_{min}\right)\wedge \left(\left|y\right|<{\mathrm{acc}}_{min}\right) \end{array}$$

$$\theta_{AP}=\{\begin{array}{ll} \mathrm{atan}2\left(z,x\right), & \mathrm{if}\;\left(x>0\right)\wedge \left(\left|y\right|>\left|z\right|-{\mathrm{acc}}_{thresh}\right)\\ 0, & \mathrm{if}\;\left(\left|x\right|<{\mathrm{acc}}_{min}\right)\wedge \left(\left|z\right|<{\mathrm{acc}}_{min}\right) \end{array}$$

The cut-off values used were ${\mathrm{acc}}_{thresh}$ = 0.1 × 9.81 m/s^2^ and ${\mathrm{acc}}_{min}$ = 0.4 × 9.81 m/s^2^.

**Figure 4:**
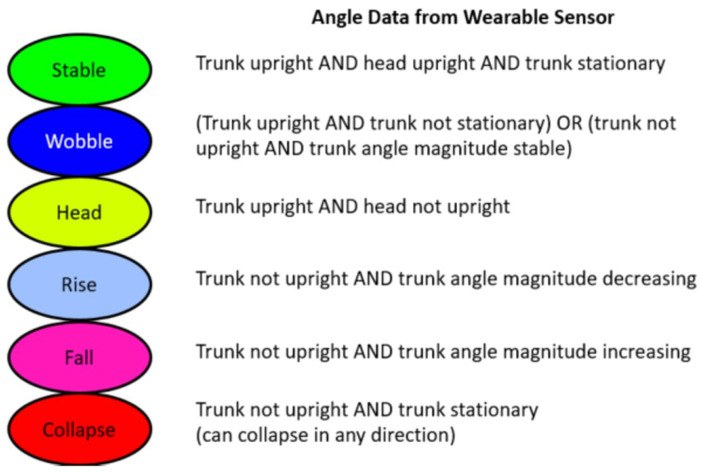
Descriptions of stages of control including color code.

Next, a time series of postural behaviors was generated using the time series data for head and trunk angles derived from the accelerometers. The behaviors classified were: Stable, Wobble, Head, Rise, Fall, and Collapse. The additional category of “Head” (not used in the initial Saavedra study) was included based on video observation that the trunk could be oriented upright while the head was not. This behavior was seen more often when external support was provided. The algorithmic descriptions of these behaviors and their color coding are shown in Figure 4.

The algorithmic descriptions and thresholds were heuristically derived and verified with video review and discussion among the first three authors (PM, SS, LTP). The values we used are expressed functionally in Table 2. These definitions were applied to the ML and AP angle time series every second to generate the time series of behaviors.

After generating the time series of behaviors, a Markov chain-like model was derived corresponding to the behavior state changes (The Saavedra et al. [39] descriptions suggested a linear sequence of change over months by aggregating the full 3-min trial into primary stages. In alignment with the increased granularity of 1-s time intervals used for the Markov chain models, behaviors are referred to as “states” rather than “stages”. The term “state” reflects moment to moment changes in behavior. The term “stage” reflects a more global phase of postural development). 

**Figure 5:**
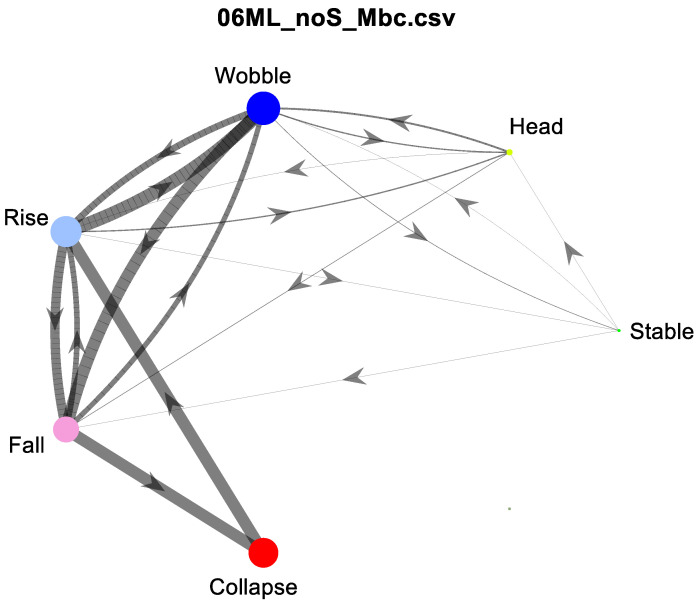
Model of postural development showing stages (colored circles), amount of time spent in each stage (relative size of circles), transitions between states (lines with arrows), and frequency of transitions coming from each node (line thickness). In this example, the child spent most time in Wobble, Rise and Fall, and Collapse and frequently transitioned from Rise to Wobble, Wobble to Fall, Fall to Collapse, and Collapse to Rise.

An example of this model is shown in Figure 5. The purpose of the model was to clearly and visually show two characteristics of the times series of behaviors: (1) how probable was each behavior transition (e.g., how likely was the transition from Stable to Wobble vs. Stable to Fall), and (2) what proportion of time was spent in each behavior.

For this model, the behaviors are abstracted in such a way that it was assumed the transition only depends on the current behavior state and not on the history of behaviors. Unlike a typical Markov chain, this study only modeled behavior changes, i.e., transitions from a behavior to another, but not to itself. As such, a transition did not necessarily occur every $t_{trans}$ seconds, but every $kt_{trans}$, where k>1, and *k* was variable over the time series. The resulting model contains transition probabilities between each state, which are indicated by the thickness of the curves between nodes in Figure 5. To generate the transition probabilities, the data were aggregated into a matrix of transition counts, which was then normalized by the total number of transitions between states as shown in Table 3. Note that the diagonal (gray cells) indicates same state transitions, which are not included in the calculation because the model only considers transitions to a different state. This matrix of values was then normalized by dividing each value by the total number of transitions between states excluding the diagonal (gray cells). Summing across each row (excluding gray cells) results in a value of one since a state must transition to some other state. Summing down each column (including gray cells) indicates relative amount of time spent in each behavior state. This is visually represented by the size of the nodes in Figure 5. The node size is proportional to time spent in the behavior state.

The model was further aggregated to generate a two-dimensional score for a given behavior time series consisting of a state score (*SS*) and a transition score (*TS*). The state score was calculated as follows:

$$SS=\frac{w_{stable}T_{stable}+w_{head}T_{head}+w_{wobble}T_{wobble}}{\max\left(w_{stable},w_{head},w_{wobble}\right)}$$

where $w_{stable}$, $w_{head}$, and $w_{wobble}$ are weights indicating the relative importance of the Stable, Head, and Wobble states, respectively. $T_{stable}$, $T_{head}$, and $T_{wobble}$ are the proportional times spent in the Stable, Head, and Wobble states respectively. The state score is normalized so that only the relative and not absolute magnitudes of the weights affect the score. That is, using weight values (2, 0.5, 1) and (4, 1, 2) will result in the same score. *SS* is a measure of how much time was spent in a “good” state (Stable, Head, Wobble) with the weights heuristically derived to indicate the relative goodness of the good states.

The transition score *TS* was calculated as follows

$$TS=\frac{w_{stable}S_{stable}+w_{head}S_{head}+w_{wobble}S_{wobble}}{\max\left(w_{stable},w_{head},w_{wobble}\right)}$$

where

$$S_{stable}=\frac{P_{rise,\;stable}+P_{fall,\;stable}+P_{collapse,\;stable}}{S_{all}}$$

$$S_{head}=\frac{P_{rise,\;head}+P_{fall,\;head}+P_{collapse,\;head}}{S_{all}}$$

$$S_{wobble}=\frac{P_{rise,\;wobble}+P_{fall,\;wobble}+P_{collapse,\;wobble}}{S_{all}}$$

$$S_{all}=\underset{\begin{array}{c} i\in \left\{rise,\;fall,collapse\right\}\\ j\in \left\{rise,fall,collapse,stable,head,wobble\right\}\; \end{array}}{\sum }P_{i,j}$$

*P_a,b_* is the probability of transition from state *a* to state *b*, and the weights $w_{stable}$, $w_{head}$, and $w_{wobble}$ are the same as described above. As with *SS*, *TS* is normalized so that it only depends on the relative values of the weights. *TS* is a measure of how often “bad” states (Rise, Fall, Collapse) transition to “good” states (Stable, Head, Wobble).

## 3. Results

Accelerometry data were collected at 128 Hz, and the algorithms used data windows of one second to determine changes in behavior. The amount of time involved in processing the algorithm was less than 30 s to classify 12 min of data. Examples are provided to indicate the improved granularity over time. The histograms that were previously used by Saavedra give a global image of postural behaviors over the 12 min; however, distinguishing fluctuations in behavior is not precise with a subjective view of the histogram.

**Figure 6:**
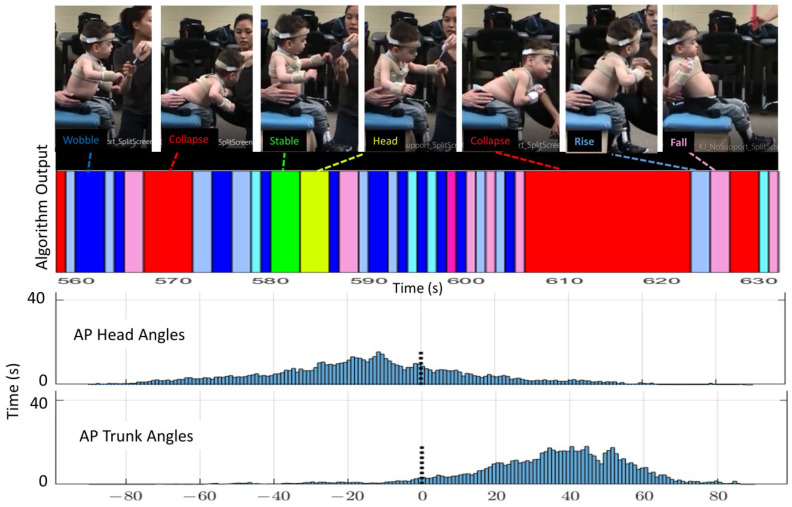
Example of algorithm output (colored bar, 70 s duration), AP histograms (bottom, full 12-min trial), and AP images from video for one child (02KJ) during the no support condition. The histogram is qualitatively most consistent with Wobble and Rise/Fall behaviors. The child’s head is more vertically aligned while his trunk is consistently leaning forward.

**Figure 7:**
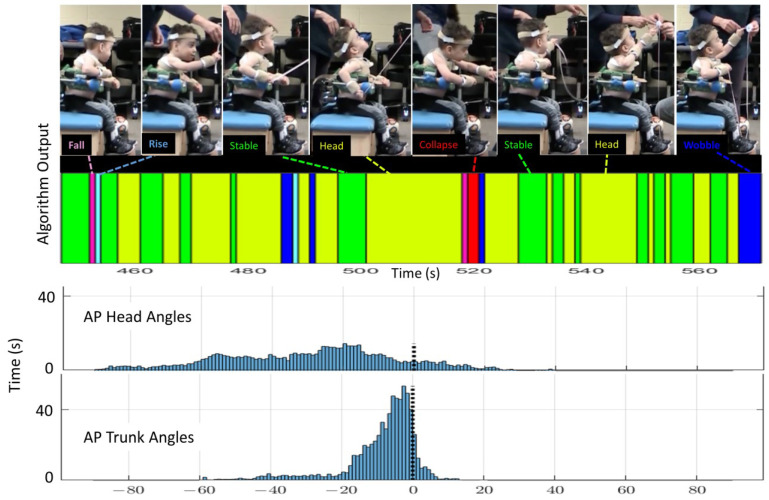
Example of algorithm output (colored bar), AP histograms (bottom) and images from video demonstrating AP alignment matched to algorithm output for subject 02KJ with mid-thoracic support. The histogram is qualitatively consistent with stable trunk and head not aligned some backward collapse is noted. For this child, the support provided a more upright and stable trunk however he tended to tip his chin upward and tilt his head back slightly.

These differences between this study’s algorithm and the histogram are illustrated in Figure 6 and Figure 7. In these figures, the colors represent the postural behavior states as shown in Figure 4. For Rise and Fall, the exact shades of the pink and light blue indicate direction of Fall or Rise (e.g., Rising from left or Falling forward). The histograms show angular position in only AP for the head and trunk across the full 12 min, whereas behavior states are determined by the algorithm using both AP and ML position and velocity. Behavior code data shown in the figure represent approximately one minute to allow visualization of the density of transitions. Figure 6 is an example of 02KJ during the no support condition. The histograms for this child primarily suggest Wobble behavior as data for the head and trunk are grossly bell-shaped and not centered around zero (vertical). There is some multimodal appearance (multiple hills and valleys) suggesting some Rise and Fall behavior. For the full 12-min data set, this study’s algorithm outcomes disagree slightly with the histogram interpretation as the algorithm quantified Wobble 25.2%, Rise 25.1%, Fall 17.1%, Collapse 30.7%, Stable 0.6%, and Head 1.3%. In retrospect, Collapse might be indicated in the histogram by noting that there was time spent leaning forward more than 60 degrees; however, 02KJ did not remain Collapsed at a specific end range so the histogram did not show a skewed distribution. Figure 7 shows the same child with mid-thoracic support and allows comparison between the histograms and output from the algorithm. The histograms at this level of support primarily suggest Stable and Head behaviors as the trunk pattern is centered closer to vertical, and, although still bell-shaped, it is narrower and steeper. There is some backwards excursion of the trunk and head on the histogram suggesting some Rise and Fall or Collapse. The histogram for the head shows the head out of alignment with the trunk, which is vertical, and, therefore, would anticipate a high percentage of behavior classified as Head. The algorithm quantified Head 48%, Stable 34.6%, Wobble 6.7%, Rise 3.6%, Fall 3.3%, and Collapse 3.9%, which is consistent with primarily Head and Stable behavior. Matching the video to the algorithm output allowed the researchers to note that Wobble most often occurred during the supported condition when the child was attempting to aim for a specific target. Moreover, the same pattern of neck extension that served this child in the no support condition led to a high percentage of trunk upright but head not upright. It can be seen from these examples that the algorithm provides increased granularity and quantification of postural data beyond what is available from the histograms or visual observation. Behavioral data for all children are shown in Table 4. It is notable that every child demonstrated the stages in different proportions; however, all stages were seen across the 12-min time period for each child and with each level of support.

The histograms can be used to estimate a primary behavioral response; however, that may or may not be an accurate image of the child’s postural control. Children fluctuated between postural behaviors throughout the 12-min period. Across all children, the algorithm demonstrated that no child used the same pattern for the entire trial. One child (04SD) used one pattern (Head) 67% of the time when offered upper thoracic support. The average percent of maximum time spent in one behavior in the no support condition was 26%, while the average percent of maximum time spent in one behavior in the support condition was 38%. 

**Figure 8:**
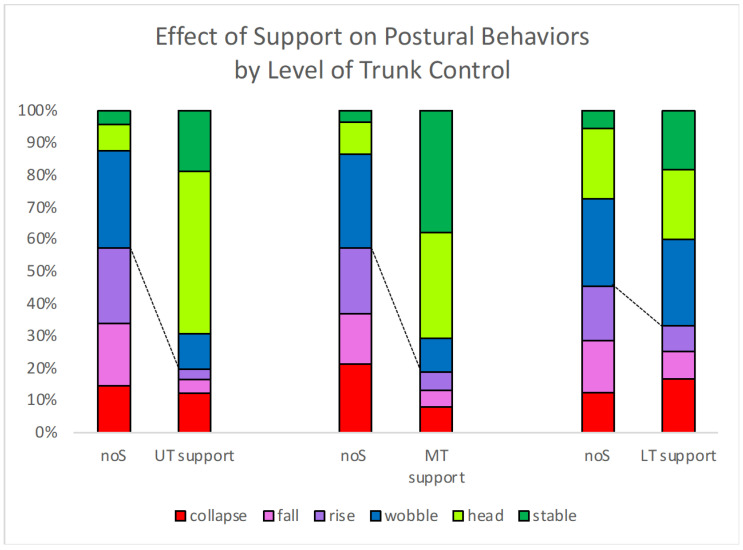
Average percent of time spent in different postural states for children who were supported at different levels of support. Dashed lines show the point of separation between poor postural behaviors (Collapse, Rise, Fall) and postural behaviors that exhibit postural response mechanisms (Wobble, Stable, Head). UT = upper thoracic, MT = mid-thoracic, and LT = lower thoracic. noS = no support.

Figure 8 shows the average percent of time spent in different postural states for children who were supported at Upper Thoracic (*n* = 3), Mid-Thoracic (*n* = 2), and Lower Thoracic (*n* = 3) levels. The change in preferred postural behaviors (Stable, Head and Wobble) was 37.6% increase for UT support, 38.4% for MT, and 12.1% for LT. Effect sizes for support were 7.4, 1.1 and 0.62, respectively. The effect size was stronger for UT because all three children improved similarly, and there was greater variation for those who were supported at MT and LT.

**Figure 9:**
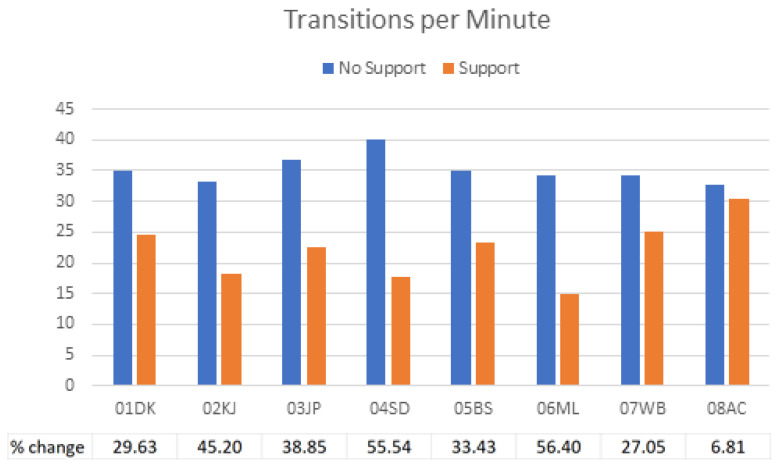
A comparison of the number of transitions per minute for each child in the unsupported and supported conditions. Each child reduced the number of transitions per minute when support was added.

Figure 9 shows how the additional thoracic support affects each child’s transitions between states. In each case, the number of transitions per minute was reduced when support was added. The greatest change was seen in 04SD and 06ML, both of whom exhibited dystonic movements and were given upper thoracic support. The smallest change was seen in 08AC who was ataxic and showed no improvement in the state and transition scores (discussed in detail below).

**Figure 10:**
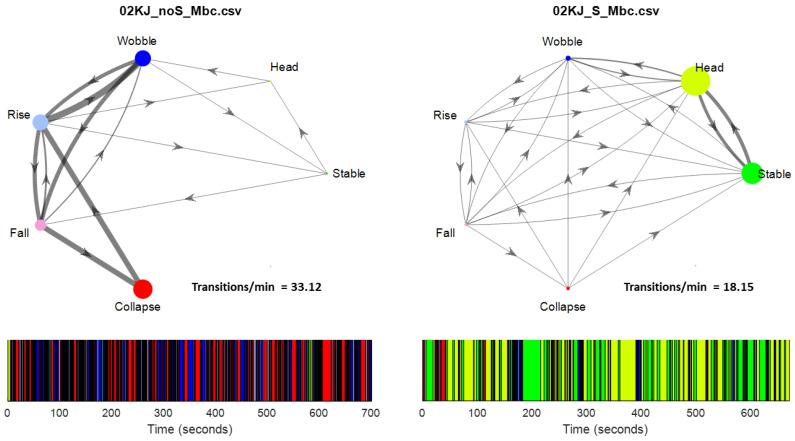
Example of the Markov model (top) and its associated behavior code time series (bottom). Left is the no support condition, and right is the support condition for the same subject. Stages (colored circles), amount of time spent in each stage (relative size of circles), transitions between states (lines with arrows), and frequency of transitions coming from each node (line thickness) are indicated in the Markov model. The transition and state scores for this child in the unsupported condition were 0.128 and 0.135, respectively. In the supported condition, the transition and state scores were 0.244 and 0.499 respectively.

Figure 10 shows the behavior state time series created by the algorithm and the resulting Markov chain model for 02KJ in both the supported and unsupported conditions. This side-by-side comparison demonstrates the differences in the child’s postural behavior when support was added. In the unsupported (pelvic strap only) condition on the left, the child spent most of the time in Wobble, Collapse, Rise, and Fall states while on the right in the mid-thoracic support condition most of the session time was spent in the Stable and Head states. This result is reflected in the state scores for this child in the unsupported and supported conditions, which were 0.135 and 0.499, respectively, and indicate significant improvement in the ability to maintain an upright posture. Similarly, the transition scores for this child were 0.128 for unsupported and 0.244 for supported conditions. These values indicate an improved ability to transition from a non-upright to an upright position. Increases in both state and transition scores indicate improved postural control.

**Figure 11:**
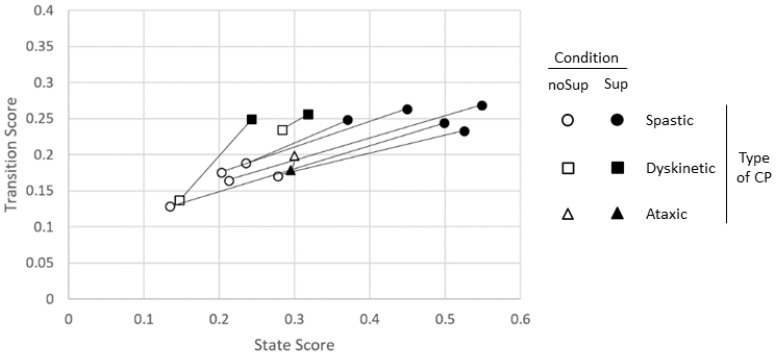
Comparison of state and transition scores for all subjects. Each subject’s no support and support score markers are connected by a line. Data points in the lower left portion of the grid indicate mostly poor posture (time spent and transitions toward Collapse or Rise and Fall), while those in the upper right quadrant indicate improved posture (time spent and transitions moving towards Wobble, Stable, or Head).

Figure 11 shows the transition and state scores for all eight participants. The algorithm was able to capture improved postural control in supported versus unsupported conditions. Improved postural control is indicated by a move upward and to the right when going from unsupported (unfilled marker) to supported (filled marker). Notably, the ataxic participant was the only one to show worse control in the supported condition compared to the unsupported.

**Figure 12:**
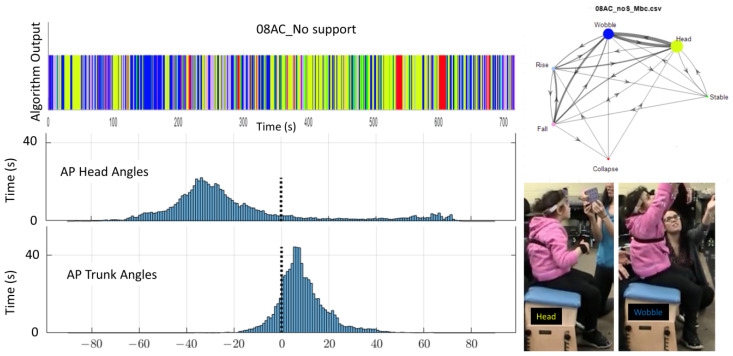
Example of algorithm output (colored bar) for the full 12-min, and AP histograms (bottom) and two images (right) showing AP alignment for 08AC during the no support condition. The histogram is qualitatively consistent with Wobble and Head. Markov model (upper right) indicates stages (colored circles), amount of time spent in each stage (relative size of circles), transitions between states (lines with arrows), and frequency of transitions coming from each node (line thickness).

**Figure 13:**
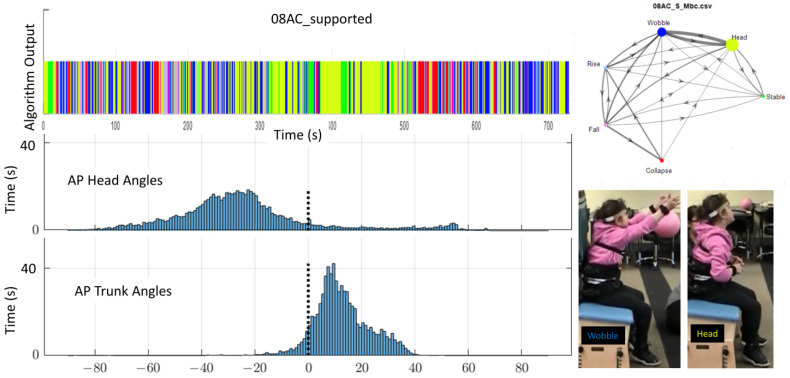
Example of algorithm output (colored bar) for the full 12 min, and AP histograms (bottom) and two images (right) showing AP alignment for 08AC during the LT support condition. The histogram is qualitatively consistent with Wobble and Head. Markov model (upper right) indicates stages (colored circles), amount of time spent in each stage (relative size of circles), transitions between states (lines with arrows), and frequency of transitions coming from each node (line thickness).

Review of video data and histogram for the participant with ataxia (08AC) supported the outcome from the algorithm that she had no apparent change (Figure 12 and Figure 13). The histograms looked similar for the two conditions. Visually, her trunk posture looked hypotonic (rounding of the spine into a c-shape) with upper trunk leaning forward and head extended for both data sets. She continued to lean her upper trunk forward and tilt her head into extension for the supported condition, even though her lower trunk and pelvis were vertically aligned. Additional sensors along the lower trunk might have been useful to document alignment of her pelvis. However, our purpose was to identify the child’s postural behavior.

## 4. Discussion

The purpose of this study is threefold. The first purpose is to determine if wearable sensors are a viable means to collect kinematic data showing orientation and stability of the head and trunk. The second is to create an algorithm that uses kinematic data to differentiate and quantify postural stages moment to moment. The sensors provided kinematic measures of the head and trunk, which were used within a custom algorithm to identify postural states as Stable, Wobble, Collapse, Fall, Rise, and Head. Histogram and video recordings were used to confirm the output of the algorithm. The third purpose is to examine transitions in postural stages across time and with different levels of support. For this purpose, a cross sectional design with children who have cerebral palsy using supported and unsupported postural conditions was implemented. The Markov chain-like model revealed that providing external support allowed all subjects: (1) to increase their time spent in the Stable state, and (2) to reduce the frequency of transitions. Furthermore, all participants except one showed improved state and transition scores when given external support, which may indicate improved postural control or, at the least, increased time spent practicing improved states of control and transition towards improved states. This is consistent with previous research that demonstrated improved trunk posture (reduced variability in trunk sway during perturbations) for typical infants (1–6 months of age) or children with cerebral palsy (GMFCS level IV or V) when increased segmental support was provided [42,49,50,51].

This algorithm is a tool that was able to quantify postural behaviors previously not captured in adult-based postural sway measures. It therefore has the potential to facilitate research and intervention for children with the most severe motor disability. The tool can test hypotheses about which state a patient may spend the most time in following an intervention. For example, an intervention focused on increasing muscle strength may be hypothesized to decrease the time spent in Collapse whereas an intervention focused on sensory feedback or reducing sensory noise may reduce the time spent in Wobble [32,51,52]. This tool can also test if subjects who more frequently transition from one particular state to other have better long-term outcomes. For example, if someone most frequently transitions straight from Stable to Fall (skipping the Wobble state), the researchers hypothesize this individual is lacking a key feedback ability to detect their own imminent fall and may have worse long-term outcomes. Finally, these findings suggest that this tool is not limited to use with CP but could be adapted for use in other populations with neurological impaired sitting ability.

Heuristic considerations were used when creating the algorithm. Parameters such as the time-interval for noting change (one second) and the limits of the cone of stability (20 degrees for trunk verticality and 30 degrees for head alignment with trunk) were selected based partly on previous postural assessments and partly from video review and reflection on the stage descriptions. The one-second time increments were selected to ensure that changes in velocity and acceleration during Rise or Fall behavior would be captured. There is some question if it is reasonable to see only one second of Wobble, or one second of Stable behavior. Most postural assessments consider only Stable upright behavior and usually set limits of 3, 5, or 10 s to be credited with postural control [23,24,53,54]. However, this study considers the development of upright control, thus examining how frequently and for how long a child is able to achieve each of these behavioral states is valuable information. One second is a reasonable option in this scenario until there is a reason to change it. The choice of 20 degrees for the trunk cone of stability was based on the amount of variation described for alignment in the Segmental Assessment of Trunk Control [26].

In this study one category was created that was not included in previous stage descriptions by Saavedra [39,40]. The category of “Head” was created based on observations of children with trunk support. Saavedra et al., 2015 [40] noted that children with cerebral palsy had more head movement than typical infants when provided with support at axillae or upper thoracic regions. With higher levels of support the trunk does not biomechanically have much range for departing from vertical alignment. Children with challenges to postural control at the level of the head or upper thoracic regions may have a more vertical trunk with support but may face challenges for head alignment. Thus, the additional category (Head) was created to indicate when the trunk is upright and the head is not aligned with the trunk. Since the trunk is allowed a range of up to 20 degrees from vertical, head alignment 20 degrees from the trunk could be a compensatory strategy to align the head and eyes vertically with the environment. However, a head angle of 30 degrees off in comparison to the trunk indicates that the head is not aligned with the trunk or the environment. The histograms (Figure 6, Figure 7, Figure 12 and Figure 13) show a common strategy adopted by children who do not have full trunk control. Forward trunk lean paired with neck extension allows them to control upright posture using only trunk and neck extensor strength. When the trunk is vertically aligned, stability requires switching between trunk flexors and extensors in a coordinated manner. Note how the child whose data are shown in Figure 6 and Figure 7 showed the trunk in front of midline and head closer to midline in the unsupported condition, however when given mid-thoracic support his trunk aligned near midline and he had a tendency to have his head fall back behind midline. This response to support suggests that the child tried to control his head using neck extensors and struggled to switch to neck flexors when needed. The other child whose data are shown in Figure 12 and Figure 13 kept the same pattern of alignment between her trunk and head under both the supported and unsupported conditions. She was given support at the lower thoracic region and thus she had more opportunity to lean her upper trunk forward in the manner she was used to. She kept her original strategy and was able to continue using primarily forward lean and neck extension to remain upright.

Data from children with cerebral palsy who are not yet able to sit independently provided a range of postural behaviors to develop this algorithm. This study’s protocol motivated children to adapt their postural behaviors over 12 min to meet the changing task requirements. It was anticipated that children might be “stuck” using a single postural behavior, so it was surprising that every child demonstrated at least a few seconds with every behavior and in only one case (04SD with support) did any behavior occur for over 50% of the time (Table 4). The activities were individualized to each child’s motivation and interest, and the same activities were repeated for the same amount of time for that child during both types of support. If a sedentary activity that did not require reaching and interacting with objects had been used, a smaller number of states and transitions might have been found. The individualized external support offered a contrast in postural challenges that allowed quantification of change in behavior for each child. Improvement in postural responses with segmental support is consistent with several previous studies examining posture and upper extremity skills [41,48,49,50]. The algorithm and histograms both reflected change in postural behavior related to change in the level of support. It was encouraging to find that the algorithm was able to show both improvement and lack of improvement with external support. Video review confirmed the algorithm classification of behavioral states.

Wearable sensors were selected for objective quantification of postural behavior because of their small size, ease of application, and potential for future use across a variety of locations. They are child-friendly and have potential scalability across time (minutes to hours to full days) and settings (laboratory, clinical, home, and community) [55,56]. Wearable sensors are used in research and as consumer products for ambulatory individuals to measure a number of physical behaviors (e.g., steps, activity level, and fall detection) [57,58]. Thus far the use of wearable sensors for non-ambulatory individuals focuses on kicking patterns in infants [55] or mobility patterns for wheelchair users [59,60]. The goal of this study was to determine if wearable sensors could provide clinically relevant information about postural control in children who were not able to sit independently. Most algorithms for documenting movement and activity with accelerometers eliminate gravitational acceleration in the outcome analyses. For this study, gravitational acceleration was used to document postural orientation and stability. While accelerometers were used for the postural data in this study, any sensor that provides head and trunk angles with respect to upright over time could be used for this type of posture behavior analysis.

Prior research into using wearable sensors to detect movement patterns in populations with mobility impairments generally use two types of algorithms [61]: (1) biomechanically-based or (2) machine learning-based. This study’s approach was to use a biomechanically-based algorithm with heuristically determined parameters, because the goal was to explain how postural control develops and not to just identify patterns. Furthermore, machine learning approaches require vast amounts of training data, which is lacking for this specific population, and the training data must be labeled. In this case, labeling would need to be done using video behavior coding, which is time intensive and challenging to accomplish. In contrast to other studies in which classifications were easily identified behaviors such as sitting, walking, stair climbing, etc., the behaviors being identified for this study are nuanced and the transitions between behaviors can occur rapidly and for brief periods of time. An unsupervised machine learning approach could overcome the lack of labeled training data; however, it would likely identify clusters of behavior that are unrelated to the biomechanical behaviors of interest.

Validity is challenging to demonstrate when measuring a construct that has not previously been studied. This study attempted a first level of validation through the confirmation of the agreement between the algorithm, histograms of AP position over time, and time matched images from video. Postural control is a continuous process. Episodes of different behaviors can be recognized as Stable, Wobble, Rise and Fall, Collapse, or Head, but it is challenging to visually distinguish the start and end points of those behaviors when a child is transitioning rapidly from one to another. The algorithm presents heuristic parameters to distinguish mathematical boundaries between these behaviors and thus provides the opportunity to quantify and analyze how the amount of time within states and the transitions between states evolve during the process of gaining upright control.

The benefit of the Markov chain model is that it can abstract the entire behavior timeseries and represent the child’s behavior as an easily interpreted visualization showing relative amounts of time spent in each behavior and the relative frequency of transitions. The state and transition scores calculated from this model can be used to compare a child’s behavior across conditions (supported vs. unsupported) or across time (months or years) to show improvement in postural control. In this study, the scores are not intended to compare postural control between different individuals.

### 4.1. Limitations

There is no gold standard measurement with which to compare the outcome of the algorithm. This study is the first attempt the authors are aware of for quantifying postural behavior states prior to development of upright control.

Data from typically developing infants were not classified or quantified in this study. It is possible that the parameters selected for children with CP may not work as well for quantification of states of control and transitions used by typical infants when gaining upright control. However, the heuristic foundations for the algorithm development came from observations of postural behavior in typical infants as well as children with CP, so it is anticipated that the algorithm will be robust to quantify behaviors across diagnostic groups and across typical or atypical developmental processes. This will require future exploration and verification.

### 4.2. Future Studies

Areas for future investigation could include:

Verification of the algorithm and model with longitudinal data from typically developing infants. Data from children who exhibit a full range of trunk control (progressing from no control to independent sitting over time) are needed to understand the links between state and transition scores and functional abilities.

Electromyography (EMG) from the trunk flexors and extensors could provide additional understanding of the developmental process. The algorithm could be used to segregate episodes of postural behavior that could be matched to the child’s EMG to examine muscle responses based on state of control. Those results could be used to understand the underlying mechanisms and suggest targets for motor learning interventions.

## 5. Clinical and Research Relevance

Use of this algorithm could provide quantification of the effectiveness of new interventions in research or in the clinic, or document upright opportunities across full day monitoring in the home and community. Lack of specificity in measurement has been paralleled by a lack of specificity for intervention and contributes to the ongoing poor prognosis for the development of functional motor skills in children with postural deficits across a variety of diagnoses. Developmental researchers have previously shown the dose and amount of experience that typical infants require for learning to walk (over 10,000 steps per day) [62]. To date, there is no information on the amount of upright experience that is required for typical infants to learn to sit independently. This method of quantifying states of control could provide benchmarks of typical development and document differences in the progression of trunk control in children with atypical development. Therapy sessions could be enhanced by providing therapists with real-time or post-session outcomes showing the effectiveness of different techniques or positioning devices during the intervention and between intervention sessions.

## 6. Patents

Patent application is pending. For more detail, see international patent publication number WO2022248939A2.

## Figures and Tables

**Table 1:** Subject Demographics. Etiologies, diagnoses, differential diagnoses and GMFCS (Gross Motor Function Classification System) levels as provided by the families’ previous participation in the Pediatric Balance Lab. Segmental Assessment of Trunk Control (SATCo) levels were determined at the start of each session. Muscle tone was confirmed for each child using the Hypertonia Assessment Tool (HAT) [48]. One child (08AC) did not demonstrate hypertonia. She had a medical diagnosis that included hypotonia and ataxia.

ID	Age (Years)	Gestational Age (Weeks)	Sex	Etiology	Diagnosis	SATCo Level	GMFCS Level	HAT
01DK	5.5	31	Male	Intrauterine asphyxia Apgar 4, respiratory insufficiency, hydrocephalus post hemorrhagic, cerebral ventricle leukomalacia, hyperbilirubinemia	Prematurity, spastic bilateral CP	Mid Thoracic	V	spasticity
02KJ	3.5	25	Male	Unknown	CP	Mid Thoracic	IV	spasticity
03JP	1.9	34	Male	Stopped moving in utero	Prematurity	Mid Thoracic	IV	spasticity
04SD	4.8	34	Female	Three small bleeds in the brainstem, apnea, low Apgar score	Prematurity, bilateral sensorineural hearing loss, dyskinetic CP	Upper Thoracic	V	dystonia
05BS	5	40	Male	Agenesis of corpus callosum	CP	Upper Thoracic	IV	spasticity
06ML	13	Overdue	Male	Unknown	Athetoid CP with dystonic movements	Upper Thoracic	V	dystonia
07WB	13	33	Male	Schizencephaly	CP	Lower Thoracic	IV	spasticity
08AC	7	23	Female	Ischemic brain injury to cerebellum, bilateral intraventricular hemorrhage (IVH)	CP	Lower Thoracic	IV	hypotonia ataxia

**Table 2:** Functional description of behaviors.

Behavior	Functional Description
Trunk Upright	The trunk (T) is considered upright if it is within a cone with respect to the vertical axis. 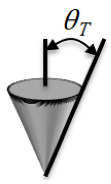 $\theta_{T}<20{}^{\circ}$
Head Upright	The head (H) is considered upright if it is within a cone with respect to the trunk axis. 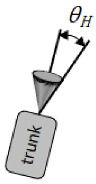 $\theta_{H}<30{}^{\circ}$
Trunk Stationary	The trunk was considered stationary at any given time if there was small variation of movement in both the ML and AP directions for an interval leading up to the current time. (Variance over 1 s < 10°)
Trunk Angle Magnitude Increasing/Decreasing/Stable	The trunk was considered to have its angle magnitude increasing or decreasing if the angular speed in the most prominent direction (ML or AP) was large. It was increasing if the angle magnitude was positive and decreasing if the velocity was negative. (Stable if slope of best fit line over 1 s < 5°/s)

**Table 3:** Raw behavior transition counts for data represented in Figure 5. The diagonal (gray shaded) cells indicate same state transitions.

		Next State					
		Stable	Head	Wobble	Rise	Fall	Collapse
**Current** **State**	**Stable**	0	1	2	0	1	0
	**Head**	0	15	11	1	3	0
	**Wobble**	3	7	122	44	82	0
	**Rise**	1	7	90	77	63	0
	**Fall**	0	0	33	37	46	78
	**Collapse**	0	0	0	79	0	148

Total Number of Transitions between States = 543.

**Table 4:** Percentages of each behavior type for each participant for both conditions. noSup = No Support, which means pelvic stability strap and manual assistance at pelvis, if necessary. MT sup = mid thoracic support, UT sup = upper thoracic support, and LT sup = lower thoracic support.

	01DK		02KJ		03JP		04SD	
	noSup	MT sup	noSup	MT sup	noSup	LT sup	noSup	UT sup
**Stable**	6.5%	41.1%	0.6%	34.6%	4.0%	18.5%	7.2%	10.9%
**Head**	18.5%	17.8%	1.3%	48.0%	22.8%	8.5%	9.6%	66.8%
**Wobble**	33.4%	14.0%	25.2%	6.7%	27.7%	33.0%	37.7%	8.5%
**Rise**	15.6%	8.4%	25.1%	3.6%	17.5%	7.9%	21.6%	3.2%
**Fall**	14.2%	7.0%	17.1%	3.3%	17.0%	6.9%	19.3%	3.6%
**Collapse**	11.7%	11.6%	30.7%	3.9%	11.0%	25.2%	4.6%	7.0%
	**05BS**		**06ML**		**07WB**		**08AC**	
	**noSup**	**UT sup**	**noSup**	**UT sup**	**noSup**	**LT sup**	**noSup**	**LT sup**
**Stable**	5.3%	43.3%	0.4%	2.6%	7.9%	29.5%	4.9%	7.3%
**Head**	11.6%	35.0%	3.2%	49.6%	5.8%	18.6%	37.1%	37.5%
**Wobble**	26.2%	5.7%	27.1%	18.6%	22.0%	21.7%	31.8%	25.7%
**Rise**	23.5%	5.1%	25.0%	1.6%	22.8%	7.1%	10.0%	9.3%
**Fall**	18.5%	6.0%	20.5%	3.6%	20.2%	8.3%	11.2%	9.9%
**Collapse**	14.9%	4.9%	23.8%	24.0%	21.3%	14.9%	5.0%	10.3%

## Data Availability

The data presented in this study may be available on request from the corresponding author. The data are not publicly available due to privacy issues related to video data.
